# Differences in the Asthma Treatment of Children Between Europe and Japan: *A Questionnaire-Based Survey Using Model Cases*

**DOI:** 10.1097/WOX.0b013e31819f20eb

**Published:** 2009-04-15

**Authors:** Mitsuhiko Nambu, Stephen Holgate

**Affiliations:** 1 Department of Pediatrics, Tenri Hospital, Nara, Japan; 2 Southampton General Hospital, Southampton, UK

**Keywords:** asthma treatment, children, Europe, Japan, questionnaire-based survey

## Abstract

**Background**

The aim of this study is to compare asthma controller therapy in children between Europe and Japan.

**Materials and Methods**

A questionnaire-based survey was conducted at the 2007 annual meeting of the European Respiratory Society held in Stockholm. In total, 120 answers were collected from European doctors. We divided Europe into 5 areas: South, West, North, East, and Central. The same survey was conducted at the 40th annual meeting of the Japanese Society of Pediatric Pulmonology. Forty-three answers were collected from Japanese doctors.

**Results**

Inhaled corticosteroids were used more frequently in Europe and antileukotrienes were used more frequently in Japan. There were also some differences in treatment in different areas of Europe.

**Conclusion**

This survey shows differences in the treatment of children with asthma in Europe and in Japan. European doctors prefer using inhaled corticosteroids, and Japanese doctors prefer using oral antileukotrienes. Because the number of the respondents is small and there may be some bias, further study on a large-scale for general clinicians providing medical care to asthma children is desirable.

## 

Some countries have their own guidelines for the treatment of children with asthma, and some use existing ones, such as the GINA guidelines. Principles of treatment of children with asthma may differ from country to country because of differences in lifestyle, economic infrastructure, religion, and so forth. To select treatment, costs should be considered in various countries and areas [1]. In Japan, the Japanese Pediatric Guideline for the Treatment and Management of Asthma was published in 2000 and has been revised every 2 to 3 years. Understanding differences in asthma treatment between countries may improve its level in that country, and also improve treatment worldwide.

In Japan, we conduct questionnaire-based surveys on the treatment and management of children with asthma every several years. The most recent survey was conducted in 2006 [2]. The surveys show the yearly tendency of asthma treatment in children, but we designed the questionnaire based on asthma severity. For example, 1 question was "What kind of treatment would you select for children with moderate persistent asthma?" We cannot use this kind of questionnaire to compare the treatment of asthma between countries because the evaluation of severity of asthma differs between countries. Therefore, we made 4 model cases of children with asthma and conducted surveys on the treatments for cases in Europe and in Japan for comparison.

## Materials and methods

The questionnaire contained 4 model cases.

### Case 1

A 14-month-old child has visited your office several times for wheezing and cough mostly with low-grade fever. He was hospitalized twice for dyspnea. His growth and development have been normal, and he has no other symptoms.

(a) If he is allergic to house dust mite, what would be your first choice of the medication(s) for his long-term controller therapy? (Multiple answers allowed.)

(b) If he does not have any inhalant allergy, what would be your first choice of the medication(s) for his long-term controller therapy? (Multiple answers allowed.)

### Case 2

A four-year-old boy is visiting your office for wheezing for the first time. He has been complaining of wheezing once or twice a month for these 3 months. In the meantime, he used his rescue beta agonists prescribed by another physician. He is allergic to house dust mite.

What would be your first choice of the medication(s) for his long-term controller therapy? (Multiple answers allowed.)

### Case 3

A 13-year-old girl is visiting your office for some chest tightness in the winter season. She felt the symptom while jogging, but it went away when she sat out for a while. She had rarely used her rescue beta agonist. Her mother noticed that she coughed several nights a week. She is allergic to house dust mite and cat hair.

What would be your first choice of the medication(s) for her long-term controller therapy? (Multiple answers allowed.)

The choice of treatment was as follows: inhaled corticosteroids (ICSs), inhaled disodium cromoglycate (DSCG), inhaled nedocromil, inhaled anticholinergics, oral antileukotrienes (LTs), oral antihistamines, oral Th2 cytokine inhibitor, oral chemical mediator release inhibitors, oral slow-release theophylline, long-acting beta agonist (inhalation or patch), no controller therapy, or others (free comment). For Case 3, "no controller therapy but beta agonists before sports only" was set up.

To make the model cases, reference was made to the examinations to become a pediatric specialist in the United States. We also asked about the background of the respondents: age, speciality (pediatrician or not?), working place (clinic, hospital, or others?), country, medications that can be prescribed, and so forth (Table 1).

**Table 1:** Characteristics of the Respondents

	No. Respondents (%)	
	
	Europe	Japan
**Age (y)**		
-29	4 (3%)	0 (0%)
30-39	31 (26%)	10 (23%)
40-49	44 (37%)	13 (30%)
50-59	35 (29%)	17 (40%)
60-	6 (5%)	3 (7%)
**Speciality**		
Pediatrics	86 (72%)	43 (100%)
**Working**		
Clinic	29 (24%)	5 (12%)
Hospital	72 (60%)	38 (88%)
Both	6 (5%)	0 (0%)

A questionnaire-based survey with these model cases was conducted at the 2007 annual meeting of the European Respiratory Society (ERS) held in Stockholm. Three pediatric asthma sessions were selected and the questionnaire was distributed at the beginning of the sessions. In total, 166 answers were collected and those of 120 European doctors were analyzed. We arbitrarily divided Europe into 5 areas. Thirteen doctors were from the South (Italy 6, Spain 5, and Portugal 2), 40 from the West (Ireland 3, England 13, The Netherlands 11, France 7, and Belgium 6), 24 from the North (Norway 10, Denmark 4, Sweden 9, and Finland 1), 21 from the East (Romania 5, Bulgaria 2, Lithuania 2, Poland 2, Latvia 2, Serbia 3, Czech 2, Croatia 1, Hungary 1, and Russia 1), and 22 from the Central (Switzerland 6, Austria 2, and Germany 14). The same survey was also conducted at the 40th annual meeting of the Japanese Society of Pediatric Pulmonology. The members of this society are mainly pediatricians. Forty-three answers were collected from Japanese doctors.

A scoring system was used. Each doctor was scored 100 points for each question. In the case of a doctor selecting several answers to each question, 100 points was divided by the number of answers: for example, when 2 treatments were selected, 50 points was given for each treatment. The points for each treatment were summed up and divided by the number of doctors. If all doctors chose only 1 treatment, that treatment was given 100 points.

## Results

### Model Case 1

#### (a) Case with house dust mite allergy (Figure 1)

ICSs scored 61 out of 100 points in Europe and 27 in Japan, whereas oral LTs scored 17 in Europe and 50 in Japan. DSCG scored more points in Japan than in Europe, although the number of points was small. ICSs scored more in the South, West, and North, and LTs scored more in the East and Central.

**Figure 1:**
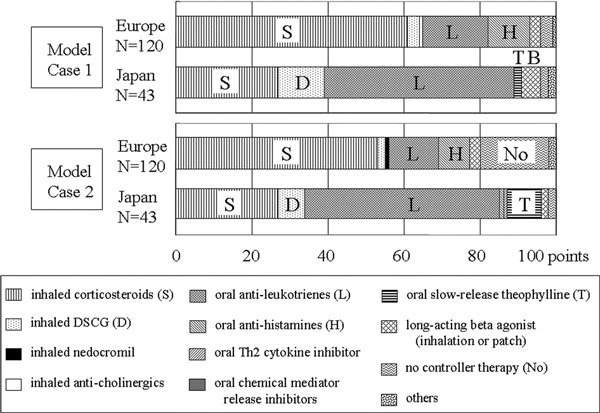
**Comparison of treatment for Model Case 1 with house dust mite allergy and for Model Case 2 between Europe and Japan**.

#### (b) Case without any inhalant allergy

ICSs scored 51 in Europe and 13 in Japan; LTs scored 25 in Europe and 57 in Japan. No controller therapy scored 15 and 6 in Europe and in Japan, respectively. ICSs scored much less and LTs scored much more in the South for this case than for the former case with house dust mite allergy.

### Model Case 2 (Figure 1)

ICSs scored 53 out of 100 points in Europe and 27 in Japan; LTs scored 13 in Europe and 51 in Japan. No controller therapy scored 18 in Europe. ICSs scored less and LTs and no controller therapy scored more in the Central than in the other areas of Europe.

### Model Case 3

ICSs scored 62 out of 100 points in Europe and 43 in Japan. DSCG scored 11 in Japan. Long-acting beta agonists scored 13 in Europe and 6 in Japan. ICSs scored less in the West and East than in the other areas. No controller therapy but beta agonists before sports only scored 14 in the West.

## Discussion

To compare asthma treatment in children between Europe and Japan, we made 4 model cases of children with asthma. The questionnaire-based surveys were conducted at the 2007 annual meeting of the ERS held in Stockholm and at the 40th annual meeting of the Japanese Society of Pediatric Pulmonology. We selected 3 pediatric asthma sessions at the ERS meeting and 86 European respondents (72%) were pediatricians (Table 1), whereas all of the Japanese respondents were pediatricians. Although there were also some other differences in characteristics of respondents between European countries and Japan (Table 1), we did not divide them into subgroups because the number of them was too small.

Because some doctors chose several answers for each question, a scoring system was introduced. This questionnaire-based survey using model cases was useful and showed the differences in the treatment of children with asthma between Europe and Japan. In general, ICSs were selected more in Europe than in Japan, and LTs were the opposite. There were also some differences shown in the areas of Europe. However, we cannot evaluate the reason for the difference because we did not ask why the treatment was chosen. Also, we should think of backgrounds of medical care systems, including insurance coverage.

In the Japanese Pediatric Guidelines for the Treatment and Management of Asthma revised in 2005, earlier introduction of ICSs was recommended. Although an increase in the usage of ICSs was observed after the revision of the guidelines in 2005 as the survey in 2006 in Japan showed,[2] ICSs may not be sufficiently propagated in Japan judging by the results of this study.

This is the first survey of its kind. Because the number of the respondents was too small and the respondents were academic meeting attendees and very much biased, further study on a large scale for general clinicians providing medical care to asthma children is desirable.

## Note

Presented at the 20th spring clinical meeting of the Japanese Society of Allergology held in Tokyo on June 12-14, 2008.

Same as above.

Sources of support: None.
